# Cell and Molecular Biology of Thyroid Disorders 2.0

**DOI:** 10.3390/ijms22041990

**Published:** 2021-02-17

**Authors:** Daniela Grimm

**Affiliations:** 1 Department of Biomedicine, Pharmacology, Aarhus University, Ole Worms Allé 4, 8000 Aarhus C, Denmark; dgg@biomed.au.dk; Tel.: +45-8716-7693; 2 Department of Microgravity and Translational Regenerative Medicine, Clinic for Plastic, Aesthetic and Hand Surgery, Otto von Guericke University, Universitätsplatz 2, 39106 Magdeburg, Germany

This issue is the second volume of the previous Special Issue, “*Cell and Molecular Biology of Thyroid Disorders*” [1].

The thyroid is a hormonal gland in vertebrates and is located in mammals on the neck below the larynx in front of the trachea [2]. It has the shape of a butterfly in humans. Its proper function is of highest importance for our health. Very often, iodine deficiency causes a malfunction of the thyroid and plays a role in carcinogenesis [3,4,5]. Low iodine intake is associated with an increased risk of thyroid cancer (TC). It increases the frequency of appearance for more aggressive histotypes [3]. In addition, both hyperthyroidism and hypothyroidism exhibit detrimental effects on the human organism.

Two articles of this Special Issue report about Graves’ disease (GD) [6,7], also known as Basedow’s disease. GD is an autoimmune disorder resulting in hyperthyroidism with an overproduction of thyroid hormones.

This hyperthyroidism is characterized by the presence of autoantibodies which stimulate the thyroid-stimulating hormone receptor (TSHR) [8,9]. The conventional therapeutic regimen (e.g., antithyroid medication, radioiodine (RAI), or surgery) still lacks efficacy for a large number of patients. The lifelong thyroid hormone replacement therapy after RAI or surgical treatment is also problematic. Therefore, novel therapeutic approaches are necessary. New options include biologics, small molecules, peptide immunomodulation, and TSHR-specific treatment modalities; specific, targeted therapies are currently under investigation [8]. 

Another disorder of the thyroid is hypothyroidism, where an underactive thyroid gland is not producing the necessary amount of thyroid hormones [1]. One form is congenital hypothyroidism (CH) which is characterized by thyroid hormone deficiency present at birth. If untreated, this disease leads to growth failure and permanent intellectual disability. Babies born with CH may be asymptomatic, or may exhibit mild symptoms that often are not recognized. 

In this issue, Kalveram et al. [10] published a research article about central CH, which is a rare and severe endocrine disorder. They demonstrated a mutation in the β-subunit of thyrotropin (thyroid-stimulating-hormone-B (TSH-B)). This mutation leads to isolated TSH-deficiency and to a severe phenotype. 

A main topic of this Special Issue is the cell and molecular biology of thyroid carcinomas. TC can occur anywhere in the thyroid gland. It is the most common type of endocrine tumours [11]. Depending on which part of the thyroid tissue the tumour originates from, TCs are classified into several categories: differentiated (DTC), including follicular (FTC), papillary (PTC) and Hürthle cell cancer; as well as anaplastic thyroid cancer (ATC) and medullary (MTC) [11,12]. The American Cancer Society estimated about 52,890 new cases of TC (12,720 in men and 40,170 in women) and 2180 deaths from TC (1040 men and 1140 women) for thyroid cancer in the United States during 2020 [13].

The majority of the thyroid carcinomas are differentiated tumours. PTC comprise about 80–90% of the TC types, whereas poorly differentiated TC (PDTC) show a frequency of approximately 10% [14]. PDTC are characterized by low-differentiation, worse prognosis, and missing response to RAI-therapy [11].

The detection of molecular gene expression changes and alterations in the proteome in TC proposes new diagnostic and prognostic molecular markers and therapeutic targets [15,16,17]. Moreover, novel drugs such as tyrosine kinase inhibitors, antibodies, and small molecules have been introduced and tested in vitro, in animals, and in clinical trials [18,19,20,21].

In this Special Issue, a total of 12 excellent papers consisting of eight original articles [6,7,10,22,23,24,25,26], and four reviews [19,27,28,29] were published, as detailed in Table 1.

This collection includes nine manuscripts focusing on TC [19,22,23,24,25,26,27,28,29], two research articles on the topic of Graves’ disease [6,7], and one article with focus on central congenital hypothyroidism [10]. The TC studies published in this issue focused on prognostic, predictive markers or biomarkers [23,24,25,27], oncogenes [22] of TC, and anti-cancer drugs [19,26,28,29].

This Special Issue covers an ex vivo study with tumour specimen of patients [23], investigating metastatic and non-metastatic samples from PTC. The transcriptome oligonucleotide microarray technology was used to detect differences between M0 and M1 PTC. Moreover, an animal study (mice) was used to the effects of *FOXE1* gene dosage reduction on cancer phenotype in vivo [22]. Single cell culture studies [10,24], in vitro studies with cell-free systems using human, rat, mouse, and rabbit hepatic microsomes [26], combined in vitro and ex vivo studies (tumour samples) [25], and single in vivo clinical studies [6,7] were included in this issue.

This Special Issue covered three studies investigating benign thyroid disorders. Graves’ disease is a very common one but with an aetiology that is still not fully understood. Polak et al. [7] investigated the relationship between the expression levels of TLR-2 and TLR-4 on CD4+ and CD8+ T lymphocytes and CD19+ B lymphocytes in patients with GD and selected clinical parameters. The authors concluded that TLR-2 and TLR-4 may serve as prognostic marker for Graves’ disease. The evaluation of peripheral blood lymphocytes expressing TLR-2 and TLR-4 suggested their important role in etiopathogenesis and clinical course of GD [7].

Another group investigated whether the Epstein–Barr Virus (EBV) plays a role in the pathogenesis of GD [6]. The authors found a significantly higher presence of EBV DNA copies in peripheral blood mononuclear cells (PBMCs) in patients newly diagnosed with GD as compared with controls. They concluded a probable role for EBV in the development of GD. EBV DNA had no effect on the severity of hyperthyroidism [6]. Central congenital hypothyroidism is a rare disorder and is caused by mutations in the β-subunit of thyrotropin (*TSHB*).

Kalveram et al. [10] characterized in vitro the pathogenic TSH β-Subunit Variant C105Vfs114X for signalling pathway modifications. They showed that this mutation induces changes in the signalling profile at the TSHR. They detected a strong decrease in cAMP signalling induction and conclude that this together with other changes in cell signalling is responsible for a severe phenotype.

Taken together, the new knowledge gained from these basic studies may impact translational research. New prognostic factors for GD or the future development of new compounds for targeting TSHR signalling are of interest.

A main focus of this issue was thyroid cancer research. Five original papers and four reviews with focus on TC were included in this issue. The transcription factor Forkhead box E1 (*FOXE1*) was investigated by Credendino et al. [22] in order to study the effects of a *FOXE1* gene dosage reduction on the cancer phenotype in vivo (mice). The in vivo mouse model demonstrated that lower *FOXE1* expression results in the development of less differentiated carcinomas, exhibiting lower proliferation and an increase in apoptosis. The authors concluded that *FOXE1* could act as a lineage-specific oncogene [22].

Another study focused on the mechanisms of distant metastases (M1) in PTC [23]. It is unclear whether there are differences in gene expression profile between metastatic and non-metastatic PTC. Further investigations are necessary with hundreds of tumours. The authors detected differences in immune-related transcripts, indicating a possible role of tumour immune infiltration for metastasis [23].

PTC are diagnosed very often and have a very good prognosis, whereas ATC are rare and have a worse prognosis [11,12,24]. Tumour Suppressor Candidate 2 (*TUSC2*) is downregulated in ATC and PTC compared to benign thyroid tissue. Mariniello et al. [24] showed in vitro that *TUSC2* overexpression reduced proliferation, migration, and the invasive potential of TC cells. In addition, TUSC2 elevated the TC cells’ sensitivity to apoptosis by induction of SMAC/DIABLO and cytochrome C proteins [24]. TUSC2 may serve as novel target and biomarker for TC.

A further study [25] investigated Prospero homeobox 1 (*PROX1*) which is involved in lymphangiogenesis. In a molecular in vitro study, a *PROX1* knockdown resulted in an increase in various angiogenesis factors, which caused an elevation in new capillary-like structures by HUVECs and upregulated focal adhesion in the tumour cells [25]. *PROX1* and *FGF2* showed opposing expression levels in FTC tissues and seven thyroid tumour-derived cell lines [25]. This interesting study demonstrated the key role of PROX1 in the spreading behaviour of TC via regulation of angiogenesis [25]. PROX1 acts as a prognostic factor and is associated with patients’ outcomes [27]. An earlier study had proposed that the reactivation of *PROX1* is a potential therapeutic strategy to attenuate disease progression in PTC [30]. Future cancer research is necessary to increase the current knowledge about PROX1 and to clarify whether it can support molecular-based treatment strategies in the clinic.

Vandetanib is a multikinase inhibitor (MKI) and used among others for the treatment of advanced MTC [31]. Indra et al. [26] published a pharmacological study investigating the microsomal metabolism of vandetanib. They identified human enzymes oxidizing vandetanib and explained the high efficiency of cytochrome P450 3A4 in the MKI’s oxidation. A review article supported this Special Issue with an update on MKI treatment (lenvatinib, sorafenib, sunitinib, cabozantinib, pazopanib, vandetanib) regarding the efficacy and safety profile in advanced refractory TC [19]. The application of these new drugs has shown favourable results in otherwise treatment-resistant TC.

Finally, the review by Varrichi et al. [28] completed this Special Issue. The authors reviewed novel data explaining how the immune system is involved in TC development and progression. In addition, cytokines are known to be involved in tumour growth and metastasis in FTC [32]. The authors discussed new results of treatment with monoclonal antibodies (mAbs) targeting immune checkpoints (IC) in patients with aggressive TCs. Monoclonal antibodies such as anti-cytotoxic T lymphocyte antigen 4 (anti-CTLA-4) or anti-programmed cell death protein-1/programmed cell death ligand-1 (anti-PD-1/PD-L1) had been used for tumour therapy, but 10% of the patients revealed a thyroid dysfunction. Therefore, combination strategies involving IC inhibitors with TKIs or serine/threonine protein kinase B-raf (*BRAF*) inhibitors are showing favourable effects in advanced TC.

Taken together, the 12 excellent publications included in this Special Issue demonstrate novel findings in the field of thyroid research.

I like to thank all the authors who supported this Special Issue. I am convinced that the application of new molecular biological technologies is helpful to improve the diagnosis and therapy of benign and malignant thyroid disorders. The detection of new biomarkers and the increasing knowledge of diagnosis, prognosis, novel targets, and new treatment strategies for TC will be important for supporting our fight against TC and contribute to reduce the mortality of advanced TC.

## Figures and Tables

**Table 1:** Contributions to the Special Issue “Cell and Molecular Biology of Thyroid Disorders 2.0”.

Author	Title	Topics	Type	Reference
Credendino S.C., et al.	Foxe1 gene dosage modulates thyroid cancer histology and differentiation in vivo	*FOXE1* behaves as lineage-specific oncogene in follicular cell-derived TC	Research Article	[22]
Szpak-Ulczok S., et al.	Differences in Gene Expression Profile of Primary Tumours in Metastatic and Non-Metastatic Papillary Thyroid Carcinoma—Do They Exist?	Valuable prognostic and predictive markers for distant metastases in PTC	Research article	[23]
Mariniello R.M., et al.	The TUSC2 Tumour Suppressor Inhibits the Malignant Phenotype of Human Thyroid Cancer Cells via SMAC/DIABLO Protein	• Tumour suppressor role of TUSC2 in thyroid carcinogenesis • promising target and biomarker for TC	Research article	[24]
Rudzińska M., et al.	Transcription Factor Prospero Homeobox 1 (PROX1) as a Potential Angiogenic Regulator of Follicular Thyroid Cancer Dissemination	in vitro study: PROX1 is involved in the spreading of TC cells by regulation of angiogenesis.	Research article	[25]
Kalveram L., et al.	The Pathogenic TSH β-Subunit Variant C105Vfs114X Causes a Modified Signalling Profile at TSHR	Central congenital hypothyroidism: • TSHB mutation C105Vfs114X • strong decrease in cAMP signalling	Research Article	[10]
Polak A., et al.	Toll-Like Receptors-2 and -4 in Graves’ Disease—Key Players or Bystanders?	Graves’ Disease (GD): relationship between TLR-2/-4 to CD4+/CD8+ T- lymphocytes and CD19+ B- lymphocytes in patients	Research Article	[7]
Indra R., et al.	Identification of Human Enzymes Oxidizing the Anti-Thyroid-Cancer Drug Vandetanib and Explanation of the High Efficiency of Cytochrome P450 3A4 in its Oxidation	The metabolism of the TKI vandetanib used for treatment of symptomatic/progressive MTC, was studied using human hepatic microsomes, recombinant cytochromes P450 (CYPs) and flavin-containing monooxygenases (FMOs).	Research Article	[26]
Pyzik A., et al.	Does the Epstein–Barr Virus Play a Role in the Pathogenesis of Graves’ Disease?	Graves’ Disease: probable role of EBV in GD development. EBV infection does not affect the clinical picture of GD.	Research Article	[6]
Rudzińska M. and Czarnocka B.	The Impact of Transcription Factor Prospero Homeobox 1 on the Regulation of Thyroid Cancer Malignancy	• PROX1 as potential prognostic marker • Its role in differentiated TC	Review	[27]
Ancker O.V., et al.	Multikinase Inhibitor Treatment in Thyroid Cancer	Multikinase inhibitors (MKIs) can be used in the treatment of advanced refractory TCs.	Review	[19]
Varricchi G., et al.	The Immune Landscape of Thyroid Cancer in the Context of Immune Checkpoint Inhibition	• Contribution of different immune cells to thyroid cancer development • Rationale for the antitumor effects of ICIs in combination with BRAF/TK inhibitors	Review	[28]
Manzella L., et al.	Activation of the IGF Axis in Thyroid Cancer: Implications for Tumorigenesis and Treatment	Role of the IGF axis in thyroid tumorigenesis update on the current knowledge of IGF-targeted combination therapies for TC	Review	[29]

## Abbreviations

ATC	Anaplastic thyroid cancer
anti-CTLA-4	anti-cytotoxic T lymphocyte antigen 4
anti-PD-1	anti-programmed cell death protein-1
anti-PD-L1	anti-programmed cell death ligand-1
*BRAF*	B-Raf (rapidly accelerated fibrosarcoma) proto-oncogene/threonine protein kinase B-
CAMP	3′,5′-cyclic adenosine monophosphate
CH	congenital hypothyroidism
DIABLO	Diablo homolog
DTC	Differentiated thyroid cancer
EBV	Epstein–Barr Virus
*FGF2*	Fibroblast Growth Factor 2
*FOXE1*	transcription factor Forkhead box E
FTC	Follicular thyroid cancer
GD	Graves’ Disease
HUVECs	Human umbilical vein endothelial cells
IC	immune checkpoints
MKI(s)	Multi-kinase inhibitor(s)
MTC	Medullary thyroid cancer
*PROX1*	Prospero homeobox 1
PTC	Papillary thyroid cancer
RAI	Radioiodine
SMAC	second mitochondria-derived activator of caspases
TC	Thyroid cancer
TKIs	Tyrosine-kinase inhibitor(s)
TSH	Thyroid-stimulating hormone
TSHB	Thyroid-stimulating hormone beta
TSHR	Thyroid-stimulating hormone receptor
*TUSC2*	Tumour Suppressor Candidate 2
